# Significant Glaucomatous Visual Field Progression in the First Two Years: What Does It Mean?

**DOI:** 10.1167/tvst.5.6.1

**Published:** 2016-11-01

**Authors:** Andrew J. Anderson

**Affiliations:** 1 Department of Optometry and Vision Sciences, The University of Melbourne, Parkville 3010, Australia

**Keywords:** visual field, progression, glaucoma, perimetry, positive predictive value, automated perimetry

## Abstract

**Purpose:**

It has been recommended that multiple visual field examinations be performed in the first 2 years after glaucoma diagnosis so that rapid visual field progression (≤−2 dB/year, using ordinary least squares regression over time of the summary index mean deviation [MD]) can be detected. Here I investigate how predictive a statistically significant regression slope is of truly rapid visual field progression.

**Methods:**

I simulated visual field series (*N* = 100,000) spaced at 4 monthly intervals for the first 2 years. MD values had a standard deviation of 1 dB. The true underlying rates of progression were selected from a modified hyperbolic secant with parameters averaged from fits to large data sets from Canada, Sweden, and the United States.

**Results:**

The positive predictive value (PPV) for rapid progression was 0.47 after 2 years, whereas the negative predictive value (NPV) was > 0.99. When using the criterion that a significant regression also had to have a slope of ≤ −2 dB/year, the PPV for rapid progression reduced substantially to 0.18 but the NPV was essentially unchanged (NPV >0.99).

**Conclusion:**

Although performing multiple visual fields in the first 2 years provides appropriate power to detect rapid progression, a significant regression slope in the first 2 years is not highly predictive of rapid progression, particularly so if slopes ≤ −2 dB/year are considered only.

**Translational Relevance:**

Statistically significant visual field progression in a short period after diagnosis may not necessarily indicate the presence of rapid progression, and so confirmatory signs of rapid progression should be sought before implementing treatment changes.

## Introduction

Estimating the rate of visual field loss in glaucoma is vital for assessing whether a patient's current treatment is adequately controlling the disease, as a large proportion of patients continue to lose vision on standard glaucoma intraocular pressure-lowering therapies.^1^ Many patients show progressive visual field loss in the absence of clear structural changes to the optic nerve head,^1^ and newer ocular imaging methods—despite their increasing use as diagnostic adjuncts—are not substitutes for visual field testing.^2^

It has been recommended that six visual field examinations be performed in the first 2 years after diagnosis so that rapid visual field progression (≤−2 dB/year, using a linear regression over time of the summary index mean deviation [MD]) can be detected.^3^ This recommendation is based on achieving a sufficient power to detect a significant rate of decline in MD when progression is rapid,^3^ and has been influential in shaping glaucoma management guidelines.^4^ However, it has been noted that whilst the *statistical significance* of the rate might be established after 2 years (i.e., the slope is significantly different from zero), the *rate* itself is poorly defined.^5^ For example, assuming moderately variable fields and a true rate of progression of −2 dB/year, slope estimates after six visual fields ranged from −0.8 to −3.2 dB/year (95% limits).^5^ Correspondingly, variability can produce rate estimates < −2 dB/year after six visual fields in people who do not have rapid visual field progression.^5^ Given a series of visual fields on a particular patient that shows a significant rate of loss and an estimated slope of −2 dB/year, what is the likelihood that the patient indeed has rapid visual field progression? Examining the distribution of estimated visual field progression rates in several large population studies shows that visual field progression rates ≤ −2 dB/year are relatively uncommon.^6^ Because of this, a significant slope estimate less than −2 dB/year may in fact be poorly predictive of rapid progression (i.e., the *positive predictive value* [PPV] may be low). This may be the case despite the power to detect rapid progression having been shown to be good,^3^ as power calculations do not consider the prevalence of rapid progression. The presumed presence of rapid progression may be a trigger for more aggressive treatments such as surgery, and surgical treatments for glaucoma have nontrivial risks of vision loss^7^ and postoperative complications.^8^ Therefore, numerically estimating the PPV for a rapidly progressing series of fields is of importance in determining how frequently rapid visual field progression is overcalled.

Simulation studies have typically examined the nature of progression rate estimates only after a 2-year period has elapsed and six visual fields have been performed.^3,5^ Linear regression can, however, return a significant value for slope after as few as three visual fields. Clinicians will examine the series of visual fields after every visit, meaning that there are several opportunities for a visual field series to be flagged as significantly progressing prior to reaching six visual fields. As such, a clinician may decide prior to 2 years that significant rapid progression has occurred and that a change in a patient's treatment may be warranted. Given such multiple assessments, might the potential to overcall rapid visual field progression increase? Nominally, this should be amenable to a simple calculation based on a multiplication of the false positive probability at each assessment. Such errors are not independent, however, and so are not simply predicted by the *P* value for significance in the linear regression analysis. For example, the likelihood of a false positive error after six visual fields will be increased if a false positive error has already occurred after five fields.

In this paper I use simulation methods to examine how predictive a statistically significant decline in a visual field series is of truly rapid visual field progression, particularly when multiple visual fields are measured in the first 2 years. I also examine how this prediction is influenced by checking for progression each time a visual field is added to a series, as might happen clinically. Finally, I examine whether criteria based not simply on the presence of significant progression but also on the magnitude of the estimated rate of progression, as commonly employed in pointwise linear regression analyses,^9^ might improve the PPV for detecting rapid visual field loss in glaucoma.

## Methods

### Simulation Details

Except where otherwise stated, the simulations presented in this paper were as follows. For each simulated patient, I created MD values for a series of visual fields spaced at 4 monthly intervals for the first 2 years, and then yearly up to 5 years. The true underlying progression rate for the patient was drawn from a modified hyperbolic secant distribution, as has been previously shown to suitably describe the distributions of glaucoma progression rate estimates.^6^ Although the distribution of true, underlying rates can never be truly known, previous works suggest that empirical estimates of distribution parameters are reasonably well defined provided the number of participants sampled is large and that there is an extended series of visual fields available for each.^10^ The modified hyperbolic secant distribution was sampled at 0.1 dB/year resolution and used previously reported average parameters from fits to large data sets from Canada, Sweden, and the United States (*n* = 2324, 583, and 587, respectively).^6^ For each field, the MD value was jittered from its nominal value (predicted from the time in the series multiplied by the underlying progression rate) using a normal distribution. Only the rate of progression was considered, and so the absolute value for the first MD in the series had no influence on the current simulation. The standard deviation of the jitter was 1.0 dB (moderate variability).^3,5,11^ Ordinary least squares linear regression was then applied to these jittered values to determine the visual field progression rate for the series of fields, in dB/year, along with a *P* value for this rate provided it was negative (i.e., visual field deterioration). The criterion for significance was *P* < 0.05. Although MD variability increases as damage in the visual field increases,^12,13^ this was not modelled as changes are typically small for the rates of change and length of visual field series investigated here (a change in standard deviation of ∼0.04 dB for every decibel of decrease in MD).^13^ Visual field series from 100,000 patients were simulated for each condition tested.

### Analyses

#### Positive and Negative Predictive Values

For the principal analyses, I determined PPVs and negative predictive values (NPVs) as a function of visual field series length. PPV and NPV values give the proportion of true positives and true negatives found using a particular criterion. They account for the prevalence of the particular condition being detected: in this case, a significant visual field progression with a rate equal to or greater than a particular threshold value. Unless otherwise stated, a visual field series was judged as having progressed significantly at a particular visit if the rate of progression had reached significance at that visit and/or at any visit prior to that (i.e., from visit three onwards). Such an assessment may be better aligned with what occurs clinically, where an assessment of possible progression is made at each visit and any management alterations enacted at the time significant progression is noted.

#### Longitudinal Consistency of Statistically Significant Slopes

I assessed the probability that, once a significant regression slope was first found, the slope would remain significant as subsequent fields were added to the series. This was assessed on data from the Rotterdam Eye Study, in which visual fields were assessed at approximately 6 monthly intervals on a group of 130 patients with primary glaucoma.^14^

## Results

**Figure 1:**
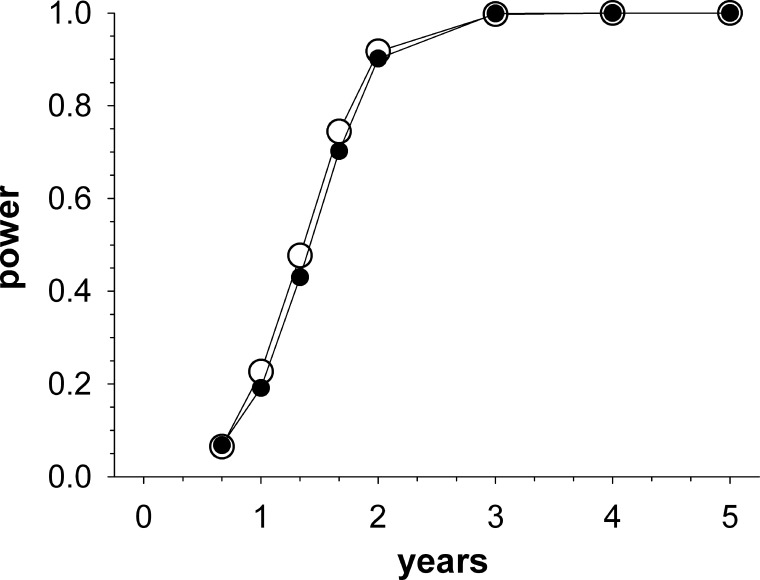
Power for detecting rapid (≤−2 dB/year) visual field progression as a function of time after diagnosis, based on the presence of a significant negative MD regression slope. *Closed circles* are for a criterion of a significant regression slope at that particular time, whereas *open circles* are for a criterion of a significant regression slope at that particular time and/or at any time prior. The simulation assumed visual fields taken at diagnosis (0 years) and at 4 monthly intervals until 2 years, and then yearly after that.

Figure 1 shows the statistical power when using a significant regression slope to detect rapid visual field progression, with the function being similar to that reported by Chauhan et al.^3^ Checking for progression at the particular time only (closed circles) returned a lower power than when progression was assessed at the time and at all times preceding it (open circles). The differences were small, however, being a maximum of 0.05 at 1.3 years and reducing to 0.02 at 2 years. Re-running both simulations resulted in alterations in power of 0.015 or less.

**Figure 2:**
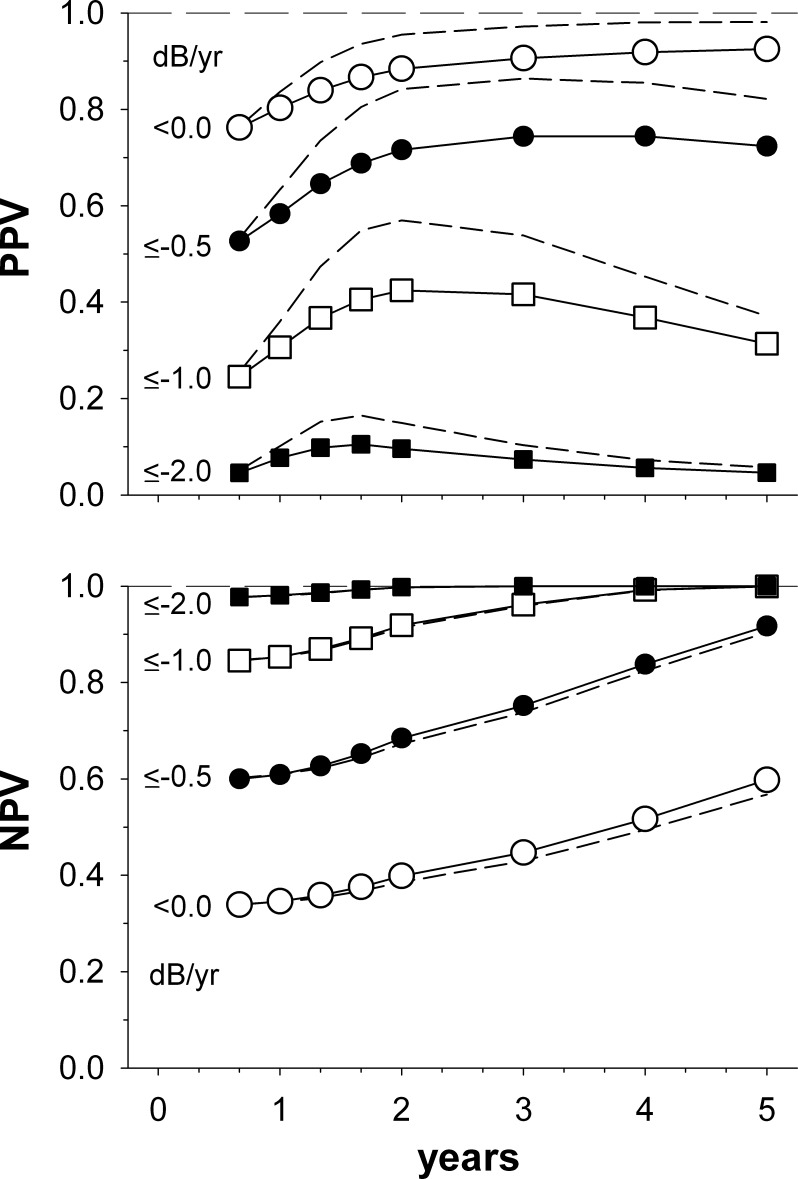
PPVs and NPVs for visual field progression worse than, or equal to, a particular rate, as a function of time after diagnosis. Visual fields were determined to be progressing at a particular time if a significant negative regression slope was found at that time, and/or at any preceding time (*symbols*). For comparison, PPV and NPV curves are shown when progression is defined as a significant negative regression slope only at the time in question (*short dashed curves*).

**Figure 3:**
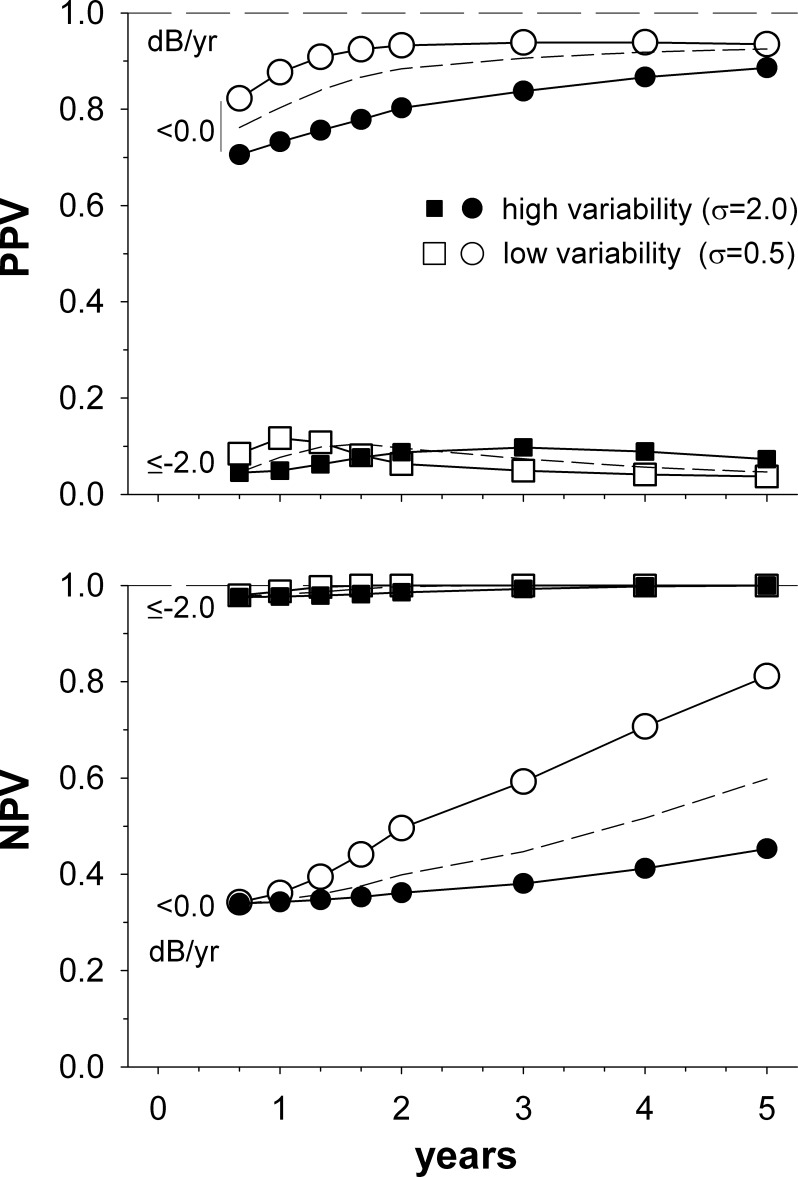
PPV and NPV for visual field progression worse than, or equal to, a particular rate, as a function of time after diagnosis. Both low (*open symbols*; σ = 0.5 dB) and high (*closed symbols*; σ = 2.0 dB) variability conditions are modelled. *Short dashed lines* show the PPV and NPV for the moderate variability condition (σ = 1.0 dB, progression at that time, and/or at any preceding time) reproduced from Figure 2. Other details are as given in Figure 2.

Figure 2 shows the PPV and NPV when the finding of a significant negative regression slope is used to detect the presence of visual field progression of a certain magnitude; for example, rapid progression of ≤ −2.0 dB/year (closed squares). Although the power for detecting rapid visual field progression at 2 years was high (Fig. 1), the PPV is only 0.47. Consequently, a finding of a significant visual field progression by 2 years is predictive of rapid visual field progression in approximately a half of cases only. NPVs for rapid progression are, however, nearly unity by 2 years, meaning that if no significant visual field deterioration is found then the probability that rapid visual field progression is truly present is very low. Indeed, even after three visual fields the NPV exceeds 97%, reflecting in large part that rapid progression is comparatively rare. For comparison, at 2 years the PPV and NPV values for detecting any progression (≤−0.1 dB/year) were 0.96 and 0.40, respectively. Assessing for progression only at a particular time (short dashed lines) improves PPV values somewhat; for example, at 2 years, the PPV for detecting rapid progression increases from 0.47 to 0.56. In contrast, NPVs alter little. For all subsequent simulations presented in Figures 3 through 5, values were calculated assuming a significant regression slope either at the nominal time and/or any time preceding it.

**Figure 4:**
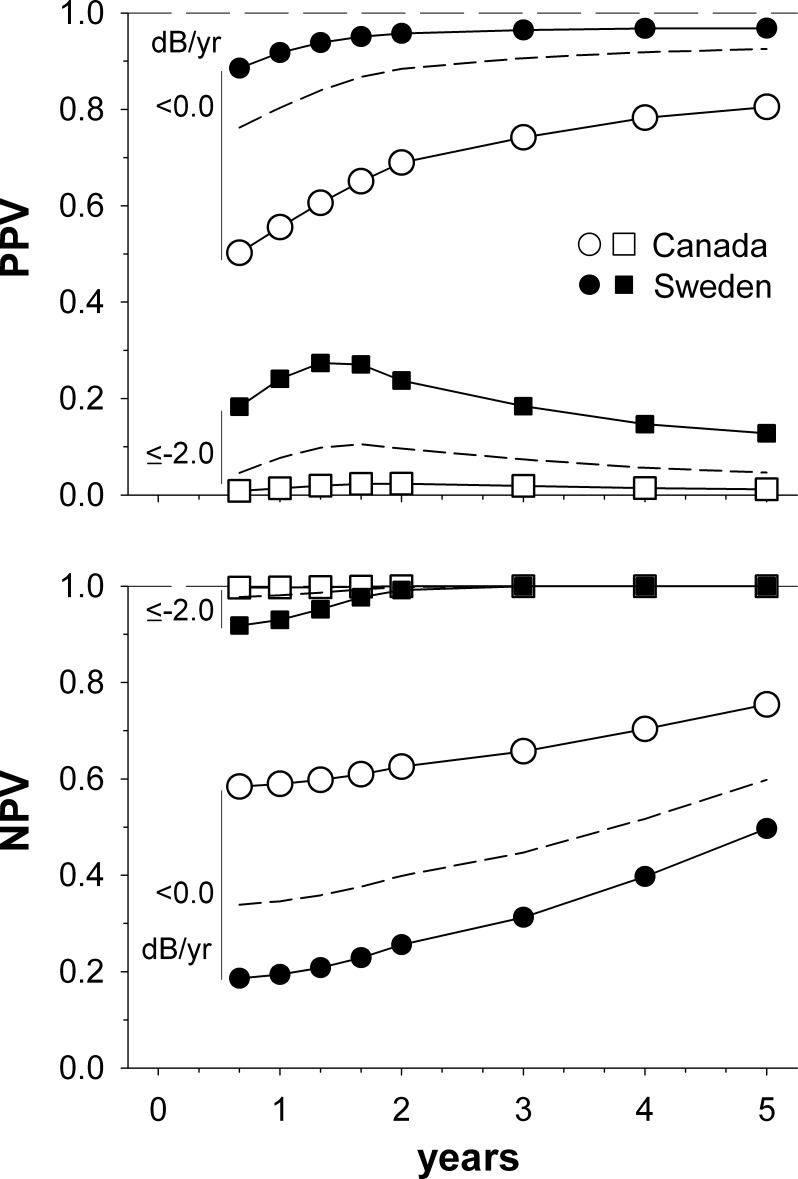
Variations in PPV and NPV for different populations. The Canadian data set included glaucoma suspects, and so showed relatively slower visual field progression than the average distribution used in the main simulation. The Swedish data set contained a large proportion of patients with pseudoexfolation glaucoma and so had a greater proportion of people with rapidly progressing visual fields than the average distribution. For comparison, the results using the average distribution (*dashed lines*; progression at that time, and/or at any preceding time) are reproduced from Figure 2. The parameters of the modified hyperbolic fits used to fit the two data sets are given in Anderson (2015).^6^ Other details are as given in Figure 2.

Visual field variability can vary between patients^3^ and between study cohorts^6^ (see also the current results from the Rotterdam Eye study, below), and is increased in patients with greater levels of field loss.^13^ It may also be decreased by the use of regression methods other than ordinary least squares regression.^15^ The influence of such variability changes is shown in Figure 3. Changing visual field variability alters where the peak PPV for rapid progression occurs, with the peak shifting to shorter times as variability reduces (Fig. 3, upper panel). Because of this, the PPV for rapid progression actually declines slightly at 2 years when visual fields are very reliable (Fig. 3, upper panel, open squares), reflecting that more visual field series with slower progression rates are able to reach significance when variability is low. As NPVs are already very high at 2 years, changing visual field variability has little effect (Fig. 3, lower panel). Figure 4 shows the influence of varying the glaucomatous population on which the simulation is run. The Canadian population (open symbols) included both frank glaucoma and glaucoma suspects, and so had a substantial proportion showing no progression and comparatively few rapid progressors.^6,16^ In contrast, the Swedish population^6,17^ (closed symbols) included a large proportion of pseudoexfoliation glaucoma patients, a disease characterized by rapid visual field progression.^18^ At 2 years, the PPV and NPV for rapid progression (squares) differed little. In contrast, the NPV for ruling out any progression (≤−0.1 dB/year; lower panels, circles) changed substantially. This difference in NPV reflects that the Swedish population had a much smaller proportion of nonprogressing patients (progression rate ≥0.0 dB/year) overall.

**Figure 5:**
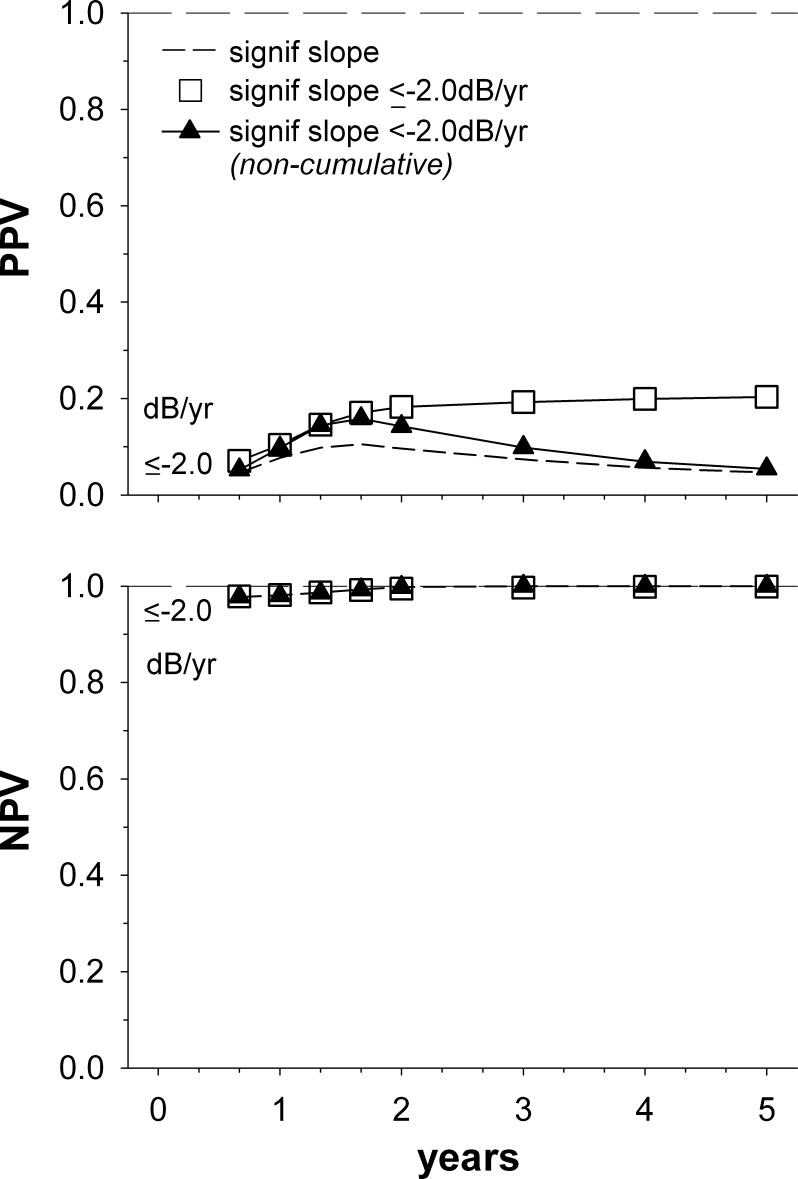
PPV and NPV for detecting rapid progression, using a criterion of either a significant nonzero slope (*short dashed line*, reproduced from Figure 2; progression at that time, and/or at any preceding time) or a significant slope that is ≤ −2.0 dB/year. For comparison, PPV is also shown for a criterion of a significant slope that is ≤ −2.0 dB/year, assessed only at the time on the x-axis (*triangles*). Other details are as given in Figure 2.

The comparatively low PPV for detecting rapid progression in Figure 2 used a criterion of a statistically significant negative regression slope, irrespective of its magnitude. Figure 5 shows the results when an additional criterion—that the slope also needed to be ≤ −2.0 dB/year—was used. The PPV dropped markedly with this additional criterion (Fig. 5, squares). Using the same criteria but only checking for progression at a particular time (triangles) produced an improving PPV over time, although at 2 years the PPV was still lower than without a slope criterion (0.46 in Fig. 5, vs. 0.56 in Fig. 2 [dashed lines]).

**Figure 6:**
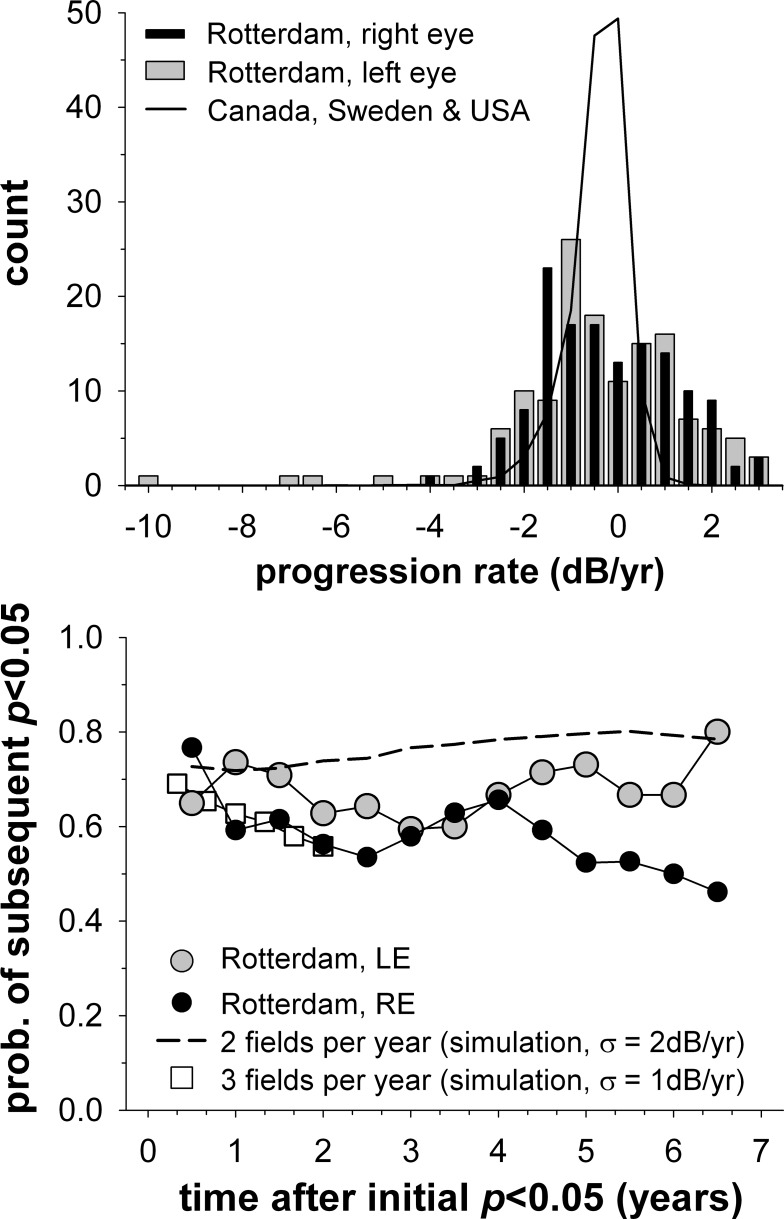
*Upper panel*: Distribution of visual field progression rates from the Rotterdam eye study, shown separately for right and left eyes (*black and gray bars*, respectively). Bars are centred on the centre of each 0.5 dB/year histogram bin. The average distribution derived from large Canadian, Swedish, and US data sets, as described in Anderson,^6^ is shown for comparison (*solid line*). *Lower panel*: Probability that a visual field series, once flagged as significantly progressing, will stay flagged as significantly progressing on subsequent visits. Only significantly progressing series were analysed (negative slopes only), with each series normalised so that the first significant progression occurred at time zero. Right and left eyes from the Rotterdam eye study were analysed separately (*black and gray circles*, respectively), with data beyond 6.5 years after first significant progression not shown as fewer than 10 visual field series existed per data point. For comparison, simulated data for highly variable visual fields tested twice a year (*dashed line*) and moderately variable fields tested three times a year (*squares*) are shown.

Figure 6 provides an insight as to why checking for progression at a particular time gives results not radically different from when progression is sought at the particular time or any time prior in the original simulation (see Figs. 1, 2). Analysis of data from the Rotterdam Eye study (lower panel, circles) shows that once a visual field series has been flagged as showing significant progression, it is likely that the series will continue to be flagged as significantly progressing as new visual field examinations are added to the series. This probability is roughly constant over time. Of note is that the distribution of progression rates in the Rotterdam Eye study (upper panel) has a large proportion of improving visual fields (positive regression slopes) compared with other studies.^6^ This suggests that visual field in this study may be more variable than in other studies. Consistent with this idea, the distribution of progression rates appears broader and more symmetric than other studies.^10^ Running a simulation using an increased visual field variability (standard deviation = 2 dB/year) produced a probability figure (dashed line, lower panel) broadly similar to that from the empirical function.

## Discussion

Performing multiple visual fields in the first couple of years after a glaucoma diagnosis provides suitable power to detect rapid visual field progression.^3^ However, knowing the power of the test alone does not allow us to assess how likely rapid progression is when a significant regression slope is found. The current study finds that the PPV at 2 years is moderate (Fig. 2), and that only approximately a half of those with statistically significant regression slopes by 2 years will have rapid visual field progression. Requiring the slope of the regression to also be ≤ −2 dB/year substantially decreased this predictive value. In contrast, other factors such as the presumed distribution of visual field progression rates (Fig. 4) and the variability of the visual fields (Fig. 3) only modestly affected the PPV.

The PPV for rapid progression did improve by approximately 0.1 by waiting 2 years before assessing for progression, rather than also assessing for progression every time a visual field was collected (Fig. 2). Although the maximum PPV was, in fact, found to be slightly earlier in our main simulation (at 20 months, Fig. 2, corresponding to six visual fields), the location of this peak alters with both visual field variability (Fig. 3) and the population distribution (Fig. 4). As such, the precise location of the peak will likely vary in practice. Based on the main simulation (Fig. 2), it could be argued that regression analyses should be avoided until performance near the peak of the PPV curve is reached—from around 16 months (five fields) given the 4 monthly testing frequency used in the current simulation. By doing this, false positive calls on rapid progression are minimized and a PPV above 0.5 is achieved.

The current simulations reveal that the PPV for rapid progression tends to fall after around 2 years, suggesting that obtaining longer series does not actually improve our ability to detect rapid progression using a criterion of a significant slope alone. Confidence intervals around regression slopes narrow as the length of visual field series increases, however.^5^ Because of this, it might be thought that an additional criterion that a steep slope (≤−2 dB/year) is present would be of increasing benefit when a large amount of visual field data is present (e.g., several years). A simulation using this criterion suggests this is not the case, however (Fig. 5, squares); the PPV remains low when a slope criterion is included, even if several years of data are obtained. This reflects the cumulative increase in false positive calls of rapid progression as the length of visual field series increases. Using a slope criterion and checking for progression at the particular time only (Fig. 5, triangles) avoids such a cumulative increase in false positive calls on rapid progression. Consequently, the PPV increases for long visual field series. Such a scenario—where for many years no regression is performed, or the regression result is not acted on—is likely not clinically realistic, however. Furthermore, at the critical 2-year mark, the PPV is still 0.1 less than if when a slope criterion is not used (Fig. 2).

Given the comparatively low PPVs described here, how might these be improved in clinical practice? Seeking confirmatory signs of rapid disease progression would be one way of improving PPVs. For example, accompanying evidence of rapid progression on regression analyses of structural imaging tests would presumably increase the likelihood that a steeply sloped visual field regression indicates rapid visual field progression. In addition, risk factors for an increased rate of visual field progression rates exist, such as increased age,^19^ increased intraocular pressure,^20^ the presence of optic nerve head changes,^21^ optic nerve haemorrhages,^22^ *β-*zone parapapillary atrophy,^20^ bilateral visual field loss,^23^ and pseudoexfoliation.^18^ The PPV should increase for patients with such risk factors, as the prevalence of rapid progression is increased for such patients. Finally, the summary index MD quantifies only the average level of visual field depression, and so also seeking spatial signs that a visual field is worsening in the way expected in progressing glaucoma would help reduce false alarms due to random noise. For example, glaucomatous visual field progression appears to occur through either the deepening of an existing scotoma or an increase in its spatial extent, rather than through the appearance of new scotomata.^24^

One aim of performing multiple visual fields early after diagnosis is to rule out rapid visual field progression, and our ability to do this is measured by the NPV. By 2 years, the NPV for rapid progression was high (≥0.99) irrespective of any manipulations of the simulation (e.g., altered variability, distribution of progression rates). Because of this, a failure to find significant visual field progression in the first 2 years is highly predictive that rapid visual field progression is not present.

Some clinicians may use the Visual Field Index (VFI), rather than MD, to assess for visual field progression. The index shows a high linear correlation with the summary index MD for visual fields with moderate loss (MD worse than −5 dB),^12^ and so it may be expected that the VFI would show similar performance to that demonstrated in the current simulations for MD. Based on this linear relationship, the rapid progression criterion of ≤ −2 dB/year corresponds to ≤ −7.3%/year in terms of the VFI. However, the index is subject to a ceiling effect where early glaucomatous visual field damage returns a VFI equal or near to 100%.^12^ Because of this, the VFI may underestimate the rate of progression in very early loss, and so the use of MD might be preferred in such circumstances.^12,25^

In summary, although performing multiple visual fields in the first 2 years provides appropriate power to detect rapid progression using regression analysis of the global index MD, a statistically significant regression slope in the first 2 years is not highly predictive of rapid progression. This is particularly the case if slopes ≤ −2 dB/year only are considered. Therefore, confirmatory signs of rapid disease progression should be sought—including via spatial information within the visual field (e.g., a consistently expanding and/or deepening scotoma)—before attempting treatment changes, particularly given that new treatments may involve surgery and its associated risks.^7,8^ To maximize the PPV, regression analysis should be avoided until around 16 months, assuming a 4 monthly test schedule, and criteria based on regression slope magnitude should be avoided. If a significant regression is not found within the first 2 years, however, the likelihood that rapid progression is present is low and so can effectively be ruled out.
